# Longevity, Age‐Specific Survival, and Mean Generation Time of *Rana muscosa* : Implications for Conservation of Possibly the Longest‐Lived Ranid Frog

**DOI:** 10.1002/ece3.72213

**Published:** 2025-10-08

**Authors:** Cynthia J. Hitchcock, Adam R. Backlin, Amanda R. Goldberg, Sarah K. Thomsen, Erin Muths, Elizabeth A. Gallegos, Robert N. Fisher

**Affiliations:** ^1^ U.S. Geological Survey Western Ecological Research Center Santa Ana California USA; ^2^ U.S. Geological Survey Western Ecological Research Center San Diego California USA; ^3^ U.S. Geological Survey Fort Collins Science Center Fort Collins Colorado USA

**Keywords:** amphibian, BaSTA, longevity, mortality, mountain yellow‐legged, survival

## Abstract

Life history strategies vary widely among species and play a vital role in extinction risk, especially in a rapidly changing environment. For many taxa, information on life history such as longevity, lifespan, and generation time is incomplete. This is especially true for amphibians, which have experienced large‐scale declines in recent decades. The mountain yellow‐legged frog ( *Rana muscosa* ) is a California endemic recognized as a state and federally endangered species. We evaluated a 23‐year dataset of six wild *R. muscosa* populations in southern California. We calculated the average lifespan of individuals in these six populations to be approximately 9.5 years, with a mean generation time of 7.4 years. We did not detect a difference in longevity between sexes or a difference in apparent survival across various ages of adults. We also documented the longest‐lived ranid frog ever recorded from a wild population: a male *R. muscosa* that was at least 21 years old. Our results suggest a relatively long generation time for this species, a characteristic that may benefit them because reproduction is regularly challenged by drought, fire activity, and disease. This information is important for understanding the complex life history of this endangered ranid frog and can help guide efforts to manage and recover the species.

## Graphical abstract

We examined 22 years of mark and recapture data for an endangered amphibian ( *Rana muscosa* ) and estimated longevity, age‐specific survival, and mean generation time. The oldest individual was determined to be at least 21 years old.

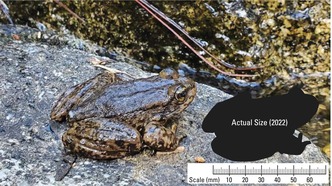

## 1 Introduction

Amphibians are declining globally at an unprecedented rate (Collins and Storfer 2003; Stuart et al. 2004; Hussain and Pandit 2012). However, threats and severity of the declines vary spatially within and among populations (Grant et al. 2020; Green et al. 2020). One particular region of concern is southern California, U.S.A., where amphibian declines have been documented for decades (Hayes and Jennings 1986; Fisher and Shaffer 1996; Lannoo 2005; Riley et al. 2005; Flaxington 2021). Despite federal protections and recovery efforts (Lannoo 2005; USFWS 2018), many species are at risk of extinction due to stressors such as habitat loss, introduced species, disease, prolonged drought, and wildfires (Griffin and Anchukaitis 2014; Swain et al. 2014; Diffenbaugh et al. 2015; Nauslar et al. 2018). Amphibians have complex life cycles with morphologically and physiologically distinct stages, and stressors can affect each stage differently (Grant et al. 2020; Nolan et al. 2023). Life history traits (e.g., length of larval stage and age at first reproduction) vary across species, and these differences can profoundly affect how species cope with environmental stressors. Linking life history information with environmental information can contribute to more ecologically relevant conservation strategies (Fonseca and Kierulff 1988; Becker et al. 2010; Amburgey et al. 2021; Liu et al. 2021). However, life history information (especially in wild populations) is incomplete for many amphibians (Mancini et al. 2025), which makes it more difficult to refine current recovery actions and tailor them specifically to individual species.

Population age structure and life history traits such as longevity and lifespan provide important information connected to reproductive strategy, survivorship, and ultimately population persistence (i.e., extinction risk; Hernández‐Yáñez et al. 2022). Lifespan in ectotherms is generally longer in cooler climates, which can be linked to higher elevations (Lai et al. 2005; Liao and Lu 2010; Oromi et al. 2012). Longevity in anurans is positively influenced by attributes such as physical or chemical protection (e.g., aposematic coloration, mimicry, skin toxins or secretions; Reinke et al. 2022). Longer‐lived species generally reproduce at a later age (Reinke et al. 2022). Likewise, body size and daily activity period can also influence longevity in amphibians, with larger or nocturnal species tending to be longer‐lived than smaller or diurnal species (Stark and Meiri 2018). Longevity may be more important than fecundity if there is a need to forgo reproduction to survive poor conditions for species living in ecosystems prone to environmental stress (Peralta‐García et al. 2022). Therefore, conservation success for longer‐lived species might be improved when focused on adult persistence and fecundity in addition to repeated reintroductions of larval or juvenile life stages.

The mountain yellow‐legged frog ( *Rana muscosa* ) is a California endemic that was once widespread but now occurs in only a few isolated locations (Backlin et al. 2013). The species is state, federally, and internationally listed as endangered (CNDDB 2025; USFWS 2002; IUCN 2022) because of declines over the last 40 years (Jennings and Hayes 1994; Vredenburg et al. 2005; Backlin et al. 2013). Historically, *R. muscosa* and *Rana sierrae* were considered the same species but were split into two different species (Vredenburg et al. 2007). Most conservation efforts have assumed that *R. muscosa* and *R. sierrae* have similar life history traits.

There are 166 documented historical populations of *R. muscosa* , but only 10 known extant populations (USFWS 2018). The remaining wild populations of *R. muscosa* in southern California inhabit headwater tributaries of mountain streams in the San Gabriel, San Bernardino, and San Jacinto Mountains (Backlin et al. 2013). Few individuals remain in each population, and natural immigration is not possible due to distances, barriers, and predatory fish between locations (Backlin et al. 2013). One study suggested the best recovery strategy is to increase the number of populations on the landscape rather than augment current populations (Chambert et al. 2022). Efforts to establish new populations through reintroduction from captive breeding populations are ongoing at suitable locations in southern California where this species is most severely imperiled (Backlin et al. 2013; IUCN 2022).

**FIGURE 1:**
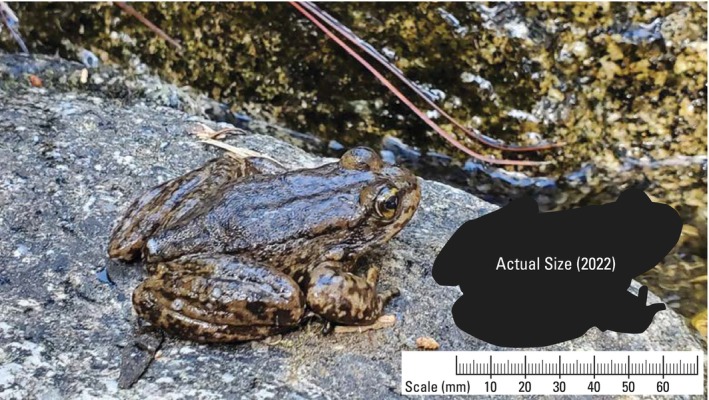
Wild‐caught male *Rana muscosa* from the Dark Canyon (PIT‐tag #: 53799366). At first capture in 2004, SVL was 65 mm and mass was 38 g. In 2022, SVL was 68 mm and mass was 37 g. Adding 3 years to reach ≥ 50 mm “adult” size, this frog is a minimum of 21 years old, but based on size at first capture, it is likely several years beyond 21. Photo credit: A.R. Backlin, USGS.

Adult *R. muscosa* are typically 40–80 mm from snout to urostyle (SUL; Stebbins and McGinnis 2012; [max = 91 mm, our data]) and are highly aquatic (Figure 1). These frogs hibernate underwater and within damp undercut banks during winter and emerge in the spring (March–April) to mate and lay eggs (Zweifel 1955; Lannoo 2005). They remain active and forage throughout the summer and fall (A. Backlin, USGS, personal observation). *Rana muscosa* tadpoles take a minimum of 2 years to metamorphose in southern California locations and are capable of reproducing one‐year post‐metamorphosis (Stebbins and McGinnis 2012, A. Backlin, USGS, personal observation). Therefore, *R. muscosa* require a minimum of 3 years to develop from egg to potentially breeding adult, which is similar to some other species of Ranidae in central and southern California ( *R. sierrae* , 4 years; *R. draytonii* , 2–3 years; *R. aurora* , 2–3 years; *R. boylii* , 2 years; Lannoo 2005). Though *R. muscosa* are diurnal and of medium body size for anurans, they possess traits that align with longer life spans, including later onset of reproduction and inhabiting high elevations where climates are cooler, often having snow for part of the year, necessitating a multi‐month hibernation period.

The U.S. Geological Survey (USGS) manages an ongoing, long‐term (2000 to present) mark‐recapture study of *R. muscosa* across southern California. We re‐examined our long‐term dataset after re‐capturing an individual in 2022 that was first marked as an adult in 2004 to address two questions: (1) what is the longevity and lifespan of *R. muscosa* in the wild, and (2) do age‐specific survival and mortality differ by site or by sex? The answers to these questions could provide new guidance on how we manage this species.

## 2 Methods

### 2.1 Study Sites

**FIGURE 2:**
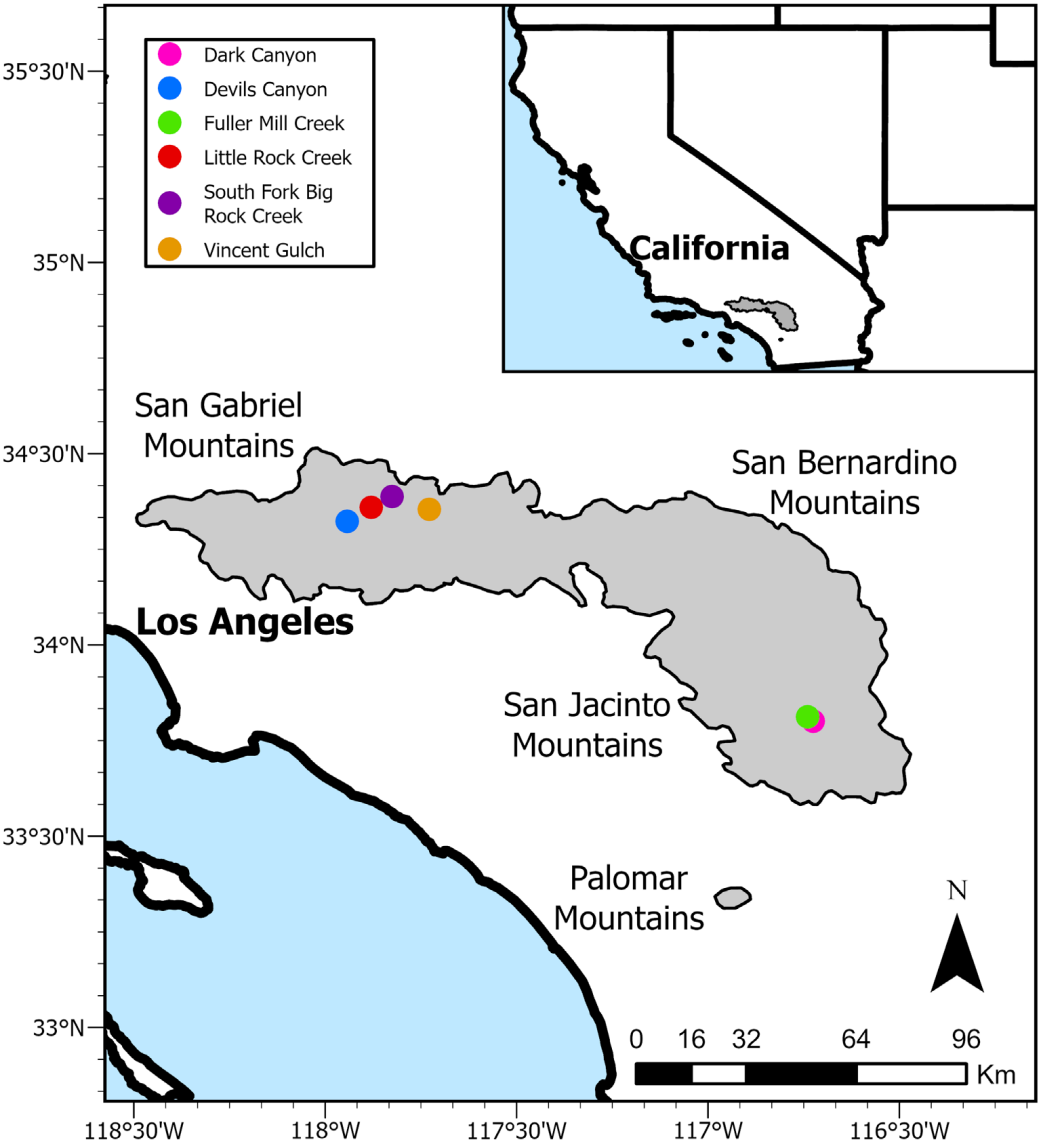
Location of the six survey sites of mountain yellow‐legged frogs ( *Rana muscosa* ) in southern California, USA. The shaded gray area represents the range of the frogs (IUCN, *Rana muscosa* . The IUCN Red List of Threatened Species. Version 2022–1). The inset shows the range (gray polygon) within the southwestern United States.

We conducted analyses using data from six sites. Our sites were located between 1615 and 1950 m in elevation, with the maximum extent of occupied habitat ranging from 1000 to 4800 consecutive linear meters of each creek (Table 1). These locations contained headwater streams with plunge pools flowing through coniferous forests and chaparral vegetation communities, typical mountain yellow‐legged frog habitat in southern California (Zweifel 1955; Lannoo 2005). Four populations are in the San Gabriel Mountains, Angeles National Forest (Vincent Gulch, Devils Canyon, Big Rock Creek, and Little Rock Creek), and two populations are in the San Jacinto Mountains, San Bernardino National Forest (Dark Canyon and Fuller Mill; Figure 2). We included only six of the 10 known extant sites in our analyses because of gaps in data at the other four locations (Table 1). Sites in our analyses did undergo a few uncommon events during the study (e.g., wildfires, post‐wildfire debris flows, and drought) that likely affected frog survival (Supporting Information—S1).

**TABLE 1:** Summary of *Rana muscosa* capture data at six sites in southern California, 2000–2022.

Mountain range	Site	Elevation (m)	Occupied habitat (m)^a^	# Individuals captured	*F*	*M*	Mean # of years individuals were captured	Standard error for mean # captures	Max age
San Gabriel	Big Rock Creek	1625	1000	141	61	77	2.65	0.16	16
San Gabriel	Devils Canyon	1900	1250	100	48	44	1.42	0.08	8
San Gabriel	Little Rock Creek	1950	2750	455	225	222	2.65	0.17	17
San Gabriel	Vincent Gulch	1615	3300	44	23	20	1.45	0.11	9
San Jacinto	Dark Canyon	1660	2250	159	54	102	2.43	0.16	21
San Jacinto	Fuller Mill	1900	4800	63	26	34	1.84	0.17	15
Total				962	437	499			
Mean		1775	2558				2.38	0.06	

*Note:* Ages represent the minimum age possible since we do not know the age at first capture. Age estimates were calculated to include a minimum of 3 years to account for 2 years of tadpole development plus another year to become an adult and receive a PIT tag (i.e., ≥ 50 mm SUL). The # of individuals captured included females (F), males (M), and individuals whose sex was never determined. We evaluated the mean number of years each *R. muscosa* was captured within each site regardless of the number of years they were known to be alive (Mean # of Years Individuals were Captured).

^a^ This is the number of consecutive linear meters of the stream that frogs occupied (i.e., length of the occupied portion of the stream).

### 2.2 Field Data Collection

Adult *R. muscosa* were highly detectable using visual encounter surveys (89% detection rate; Backlin et al. 2013). Therefore, we conducted visual encounter, capture‐mark‐recapture surveys at six sites from 2000 to 2022. We collected data on three to seven occasions per year during the active season of the frog (June–October in southern California; Stebbins and McGinnis 2012). We captured frogs by hand or with a dip net under IACUC protocol number USGS_IACUC#230410bWERCRF. We measured captured frog's SUL with a plastic ruler (±0.5 mm), determined mass with a spring scale (±0.5 g), and determined sex based on the presence or absence of nuptial pads (Zweifel 1955). We categorized frogs ≥ 50 mm as potentially breeding adults (Stebbins and McGinnis 2012). We used passive integrated transponder tags (PIT; Avid 12 or 8 mm; Dodd 2009) to uniquely mark individual adult frogs. We scanned captured frogs with a PIT‐tag reader to determine if they were new individuals or recaptures. All individuals were released at the point of capture after processing (generally within 1–5 min of capture). We assumed the minimum age of animals marked for the first time was 3 years because of their life history described above. All surveys were conducted under our federal 10(a)(1)(a) recovery permit TE‐045994‐20. Only permitted individuals handled frogs to mark, scan, and collect data on mass and length.

### 2.3 Data Analyses

We used program R v4.1.3 or v4.4.1 for analyses (R Core Team 2024), and figures were created in the “ggplot2” package (Wickham 2016). We collapsed all visits within a year to a single capture record for our analysis of annual survival/mortality rates.

#### 2.3.1 Age‐ and Sex‐Specific Survival and Mortality

We used Bayesian survival trajectory analysis to estimate age‐specific survival and mortality rates and assess differences by sex with the BaSTA package in the program R (BaSTA v 2.0; Colchero et al. 2012). This approach enables estimation of age‐specific survival even with unknown births (left truncation) and unknown deaths (right censored), and it is robust to uncertainty in ages with capture‐mark‐recapture data (Colchero and Clark 2012). First, we pooled all six sites and sexes together. We used the life table produced by BaSTA in this model (Table S1) to estimate longevity (number of years from the age of first reproduction to when 95% of individuals have died; Reinke et al. 2022), lifespan (the span of ages for a population), and life expectancy (average remaining years of life after a given age). Next, we ran models to compare differences in lifespan and mortality rates by sex at the three sites with the most PIT‐tagged individuals (Dark Canyon, Big Rock Creek, and Little Rock Creek). We ran four Markov chain Monte Carlo simulations for all models with 50,000 iterations each, with a burnin of 5001 and thinning set to 50. Recapture probability was allowed to vary each year, and we set the minimum age at three. We selected the Gompertz model of mortality, which has only two parameters: b0, which describes the basal mortality rate, and b1, which describes the exponential increase in mortality with age. We chose to use the Gompertz model over other options because our dataset is relatively sparse and the simpler approach also makes it more directly comparable to other studies (Reinke et al. 2022). Chains from all models were visually inspected along with an assessment of the diagnostic calculations of the potential scale reduction factor within BaSTA that indicate convergence. We used the Kullback–Leibler divergence calibration (KLDC) to assess the effect of sex as a covariate at three of the sites with the largest populations (Colchero et al. 2012). KLDC values closer to 1.0 on a scale from 0.5 to 1.0 indicate differing posterior distributions and therefore an effect of the covariate on mortality rates (McCulloch 1989). We considered values > 0.85 as indicating substantial differences.

We estimated age‐specific apparent survival for all the sites combined (*n* = 936 individuals) using Cormack‐Jolly‐Seber models (CJS) in MARK (White and Burnham 1999) using the package “RMark” (Laake 2013) with “time since marking” as a proxy for age. Our data comprised 23 years (sessions). We removed any individuals for whom we could not identify their sex (*n* = 26; Table 1). We used AICc model selection (Burnham and Anderson 2002) to find the best‐fit model for the probability of capture (*p*). We assumed any model with a delta AICc value under 2.0 to be competitive. We compared 14 different combinations of sex, site, and time for *p* (Table 2). We were only interested in evaluating apparent survival (Phi) by age, so all models for Phi included age only. We fixed *p* to zero for any sessions at a site that were not conducted that year (Table S2). Additionally, we fixed Phi to zero for age ≥ 21 because 21 was our oldest recorded individual. We used estimates of Fletcher *c*‐hat to assess the goodness‐of‐fit of the most global model [Phi(age) *p*(sex × time × site)] (Fletcher 2012; White and Cooch 2017).

**TABLE 2:** Model selection results for Cormack‐Jolly‐Seber models fit to capture‐mark‐recapture data from *Rana muscosa* at six sites in southern California from 2000 to 2022.

Model	K	AICc	ΔAICc	w_*i*_	Deviance
Phi(~age)*p*(~time × site + sex)	145	4653.30	0.00	0.99	2303.17
Phi(~age)*p*(~site × time)	144	4663.51	10.21	0.01	2315.69
Phi(~age)*p*(~sex × site + time)	51	4668.65	15.35	0.00	2525.19
Phi(~age)*p*(~sex + site + time)	46	4682.20	28.90	0.00	2549.27
Phi(~age)*p*(~time + site)	45	4692.32	39.02	0.00	2561.42
Phi(~age)*p*(~sex × time + site)	67	4709.31	56.01	0.00	2531.99
Phi(~age)*p*(~sex × site)	30	4746.14	92.84	0.00	2646.33
Phi(~age)*p*(~sex + time)	41	4760.77	107.48	0.00	2638.21
Phi(~age)*p*(~time)	40	4766.650	113.35	0.00	2646.16
Phi(~age)*p*(~site)	24	4767.84	114.54	0.00	2680.35
Phi(~age)*p*(~sex × time)	62	4788.38	135.08	0.00	2621.70
Phi(~age)*p*(~sex × time × site)	270	4845.43	192.13	0.00	2188.34
Phi(~age)*p*(~sex)	20	4856.05	202.75	0.00	2776.73
Phi(~age)*p*(~1)	19	4862.69	209.39	0.00	2785.41

*Note:* All models included age as the only covariate of interest for apparent survival (Phi), while we tested multiple models for the probability of capture (*p*), which included sex (male vs. female), site, and time (each year of the study). The number of parameters (*k*), Akaike information criterion score corrected for sample size (AICc), and the change in model fit relative to the top model (ΔAICc), the model weight (*w*_*i*_), and the deviance are included.

#### 2.3.2 Mean Generation Time

We also calculated the mean generation time (i.e., average age of reproductive adults) using the equation (Fung and Waples 2017; Bird et al. 2020):

(1) $$1/\left(1–\text{survival rate}\right)+\mathrm{age}\;\mathrm{at}\;\text{maturity}$$

To calculate the value to use for “survival rate” in determining mean generation time (Equation 1), we used an additional CJS model where Phi was set as constant (no difference relative to time, sex, or age), and *p* was the interaction between time and site and the additive effect of sex. Including both sexes in this calculation, versus only females, did not change the results (data not shown), improved our sample size, and results were similar to those reported by Fung and Waples (2017) for generation length. Because there was little difference between survival rates of the sexes, we included data from both sexes to improve our sample sizes. We also acknowledge that Equation 1 is only appropriate for estimating generation time if fecundity is assumed not to change with age after first reproduction, and mortality rates are constant (Maida et al. 2018).

## 3 Results

### 3.1 Capture Results

**FIGURE 3:**
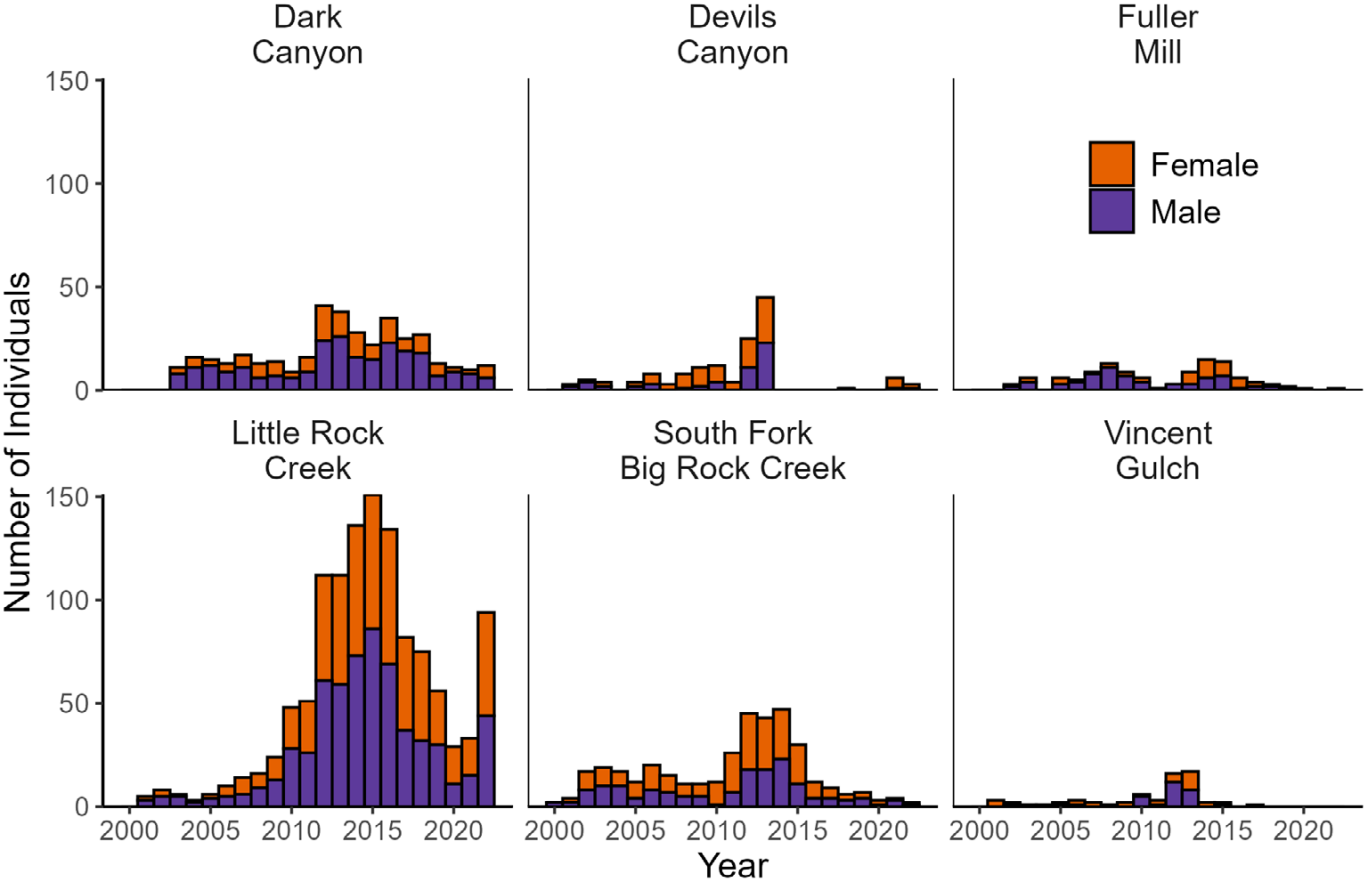
Number of individual *Rana muscosa* captured from 2000 to 2022 in California, USA at six sites.

We PIT‐tagged 962 individuals across six sites (Table 1). We captured a relatively even number of males (*n* = 499) and females (*n* = 437) within each study site, except Dark Canyon, which was male‐skewed (Table 1; Figure 3). The mean number of years in which individual frogs were captured throughout the course of the study varied depending on the site (Table 1). Each year that a frog was captured or not captured was coded as a binary number (e.g., 1 if the frog was captured at least once and 0 if the frog was not captured). Many frogs were never recaptured after being PIT‐tagged (42.8%, *n* = 377; excluding those that were tagged in the final session of the study [*n* = 81], for whom there was no possibility of recapture). We captured the most individuals at Little Rock Creek (Table 1, Figure 3). All sites appeared to experience at least one severe decline in abundance at some point during the study (Figure 3).

Average adult *R. muscosa* SUL was 59.71 mm for males (range = 50–78 mm) and 67.34 mm for females (range = 50–91 mm). Average adult body mass was 26.48 g for males (range = 10–58 g) and 38.82 g for females (range = 10–98 g).

### 3.2 Lifespan and Longevity

**FIGURE 4:**
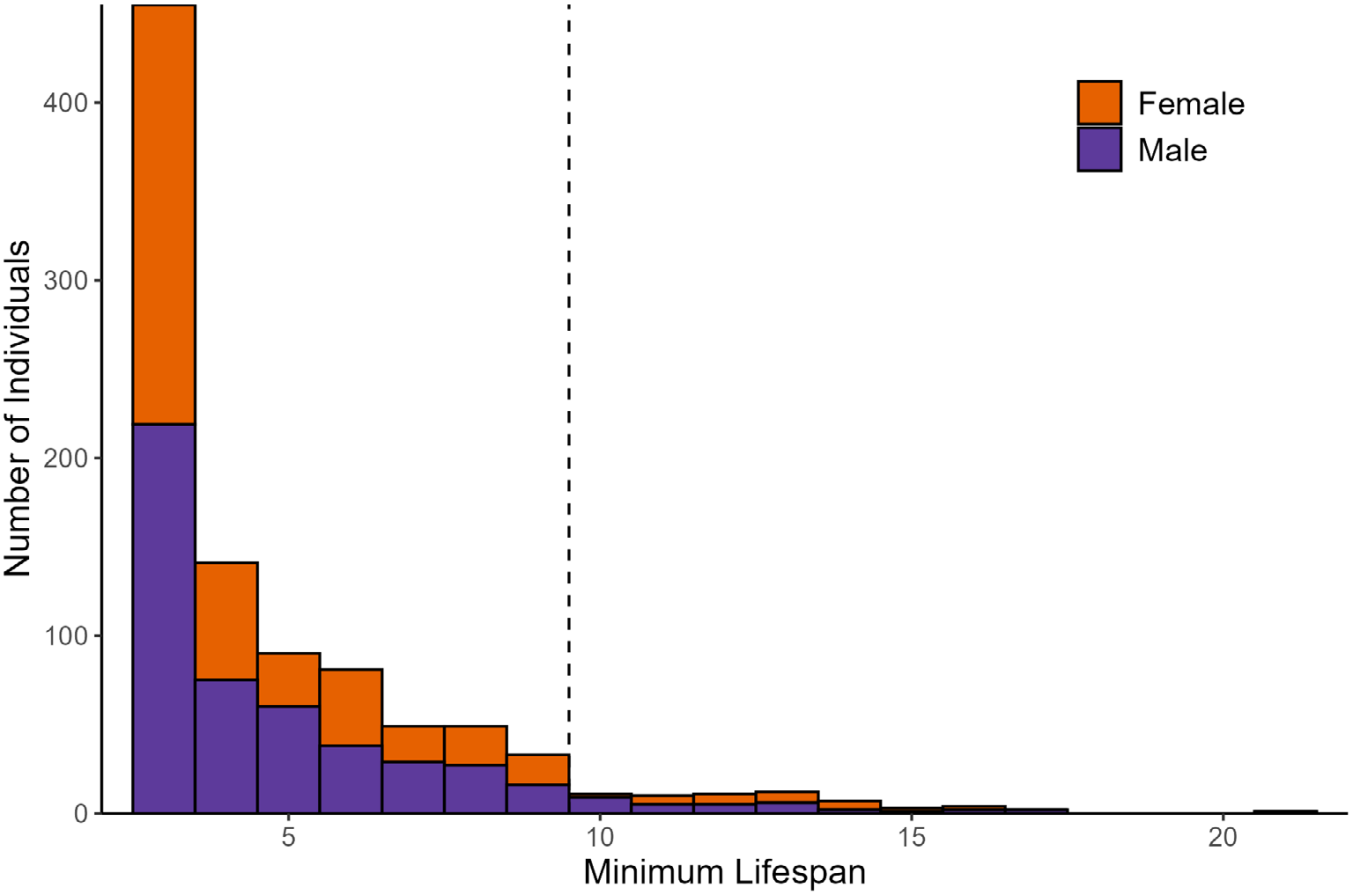
Number of individual *Rana muscosa* captured from 2000 to 2022 in California, USA. We calculated the minimum lifespan as the number of years plus three (all individuals were adults and hence had to be at least 3 years of age at first capture) that an individual lived. Age at first capture was unknown, so the actual lifespan of the individual may have been longer. The vertical dotted line represents the estimated longevity (computed as the number of years after the age at first reproduction until 95% of the population has died).

Lifespan ranged from three to 21 years (mean = 4.90 ± 0.089 SE; Figure 4). The oldest individual captured was a male at Dark Canyon who was at least 21 years old at the time of his last capture in 2022. There was no indication of senescence in 2022, but he was not subsequently captured. At first capture in 2004, the individual measured 65 mm SUL and 38 g. At his last capture in 2022, he measured 68 mm SUL and 37 g. Sixty‐one individuals across six sites were over the age of 10 (28 females and 33 males; 6.54% of the total captured individuals; Figure 4), and at two sites, no individuals were over the age of 10 (Devils Canyon and Vincent Gulch; Table 1). Big Rock Creek had the highest percentage of individuals over 10 years old (10.2%), followed by Little Rock Creek (6.9%), Fuller Mill (3.2%), and Dark Canyon (also 3.2%). We estimated that the longevity of the species was ~9.5 years across our six locations (Figure 4).

### 3.3 Age‐ and Sex‐Specific Survival and Mortality

**FIGURE 5:**
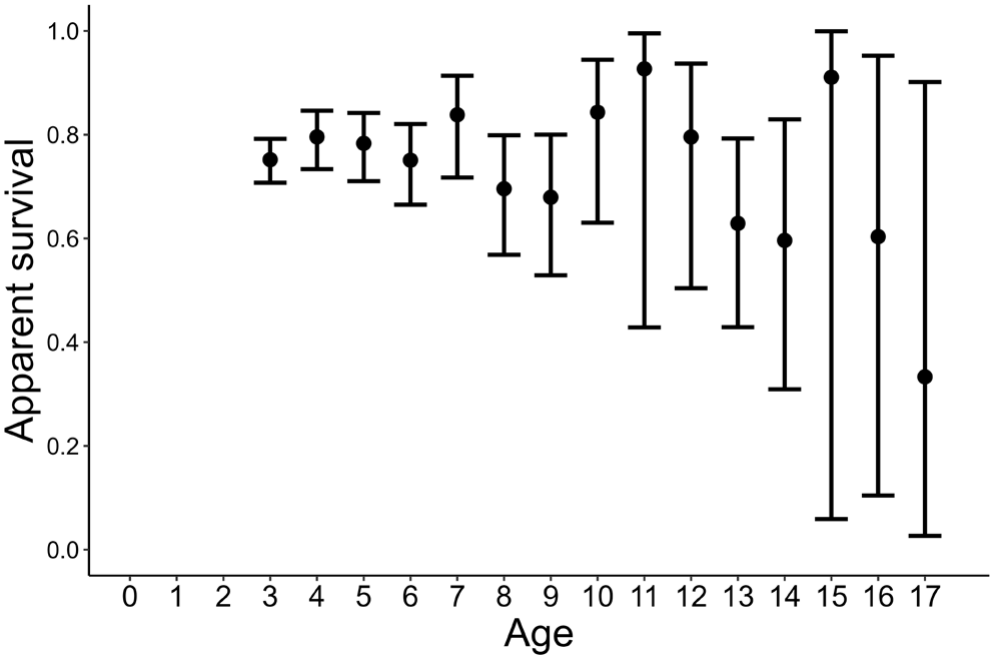
Age‐specific apparent survival rates of *Rana muscosa* captured from 2000–2022 in California, USA, using a Cormack‐Jolly‐Seber model. These estimates were obtained from the model: Phi(time since marking) *p*(time + sex × site). Error bars represent 95% confidence intervals. We assigned age three to all newly captured adults, as that is the minimum age an adult frog can be.

We did not detect a difference in apparent annual survival among ages (Figure 5). Point estimates for apparent annual survival were above 60% through age 15. The top model for the probability of capture included the interaction between time and site, plus the additive effect of sex (Table 2; Figure S1). Additionally, the Fletcher *c*‐hat for the most global model was close to 1.0 (*c*‐hat = 1.000003), so we did not account for overdispersion.

**FIGURE 6:**
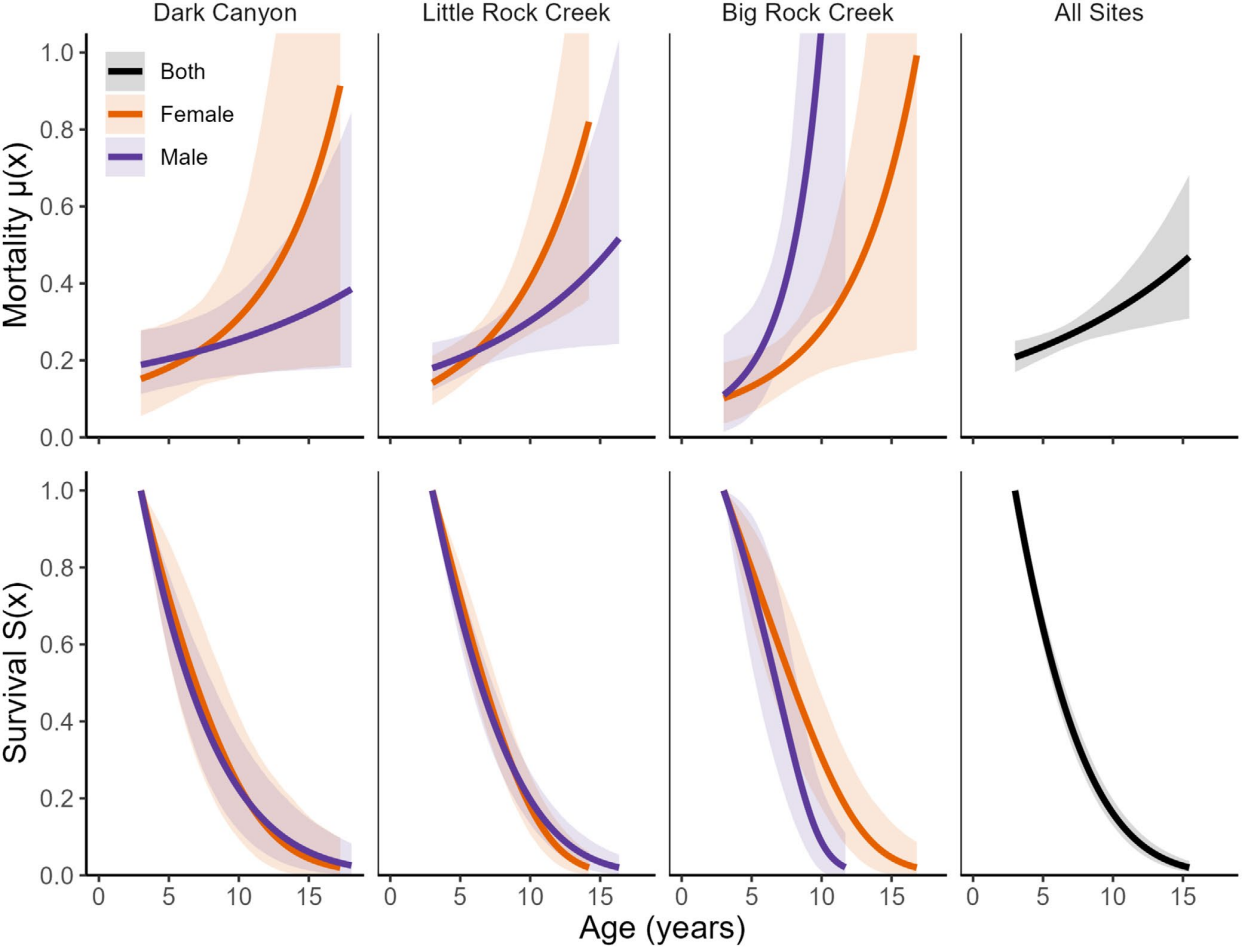
Age‐specific mortality and survival rates estimated with Bayesian survival trajectory analysis differed by site and by sex. We evaluated differences in sex at only the three sites with the largest number of individual captures. All six sites were combined (All Sites) into a single model of age‐specific mortality and survival rates (black lines). Shaded areas display the 95% credible intervals around the mortality (i.e., hazard) rate but are clipped at 1.0 to show differences more consistently at the lower bounds.

We estimated little difference in baseline mortality between sexes for the three sites with the greatest numbers of PIT‐tagged adults (Gompertz baseline mortality parameter b0; Table 3). However, there was a difference for the Gompertz parameter b1 (exponential increase in mortality rates with age), as indicated by KLDC values > 0.85 for difference between sexes (Table 3). Specifically, mortality increased with age at a higher rate for females than males at Dark Canyon and Little Rock Creek (Table 3, Figure 6), but the opposite relationship (mortality increased at a higher rate for males than females) was evident at Big Rock Creek (Table 3, Figure 6).

**TABLE 3:** Parameter mortality rates were estimated using the “BaSTA” package for the three most populous sites in southern California.

	Estimate	95% CI*	KLCD**
*Dark Canyon*			
b0.SexFemale	−1.96	(−2.89, −1.28)	0.7592
b0.SexMale	−1.70	(−2.18, −1.28)	
b1.SexFemale	0.11	(0.00, 0.28)	**0.8856**
b1.SexMale	0.04	(0.00, 0.12)	
*Little Rock*			
b0.SexFemale	−1.98	(−2.47, −1.55)	0.7742
b0.SexMale	−1.73	(−2.11, −1.40)	
b1.SexFemale	0.15	(0.06, 0.25)	**0.9026**
b1.SexMale	0.07	(0.01, 0.15)	
*Big Rock*			
b0.SexFemale	−2.38	(−3.33, −1.64)	0.6237
b0.SexMale	−2.42	(−4.22, −1.33)	
b1.SexFemale	0.15	(0.02, 0.31)	**0.9041**
b1.SexMale	0.33	(0.06, 0.71)	
*All six sites combined*			
b0	−1.57	(−1.77, −1.38)	
b1	0.06	(0.019, 0.11)	

*Note:* We calculated 95% credible intervals (CI*). All Kullback–Leibler divergence calibration (KLCD**) values > 0.85 are in bold to indicate substantial differences. (b0 = basal mortality rate; b1 = exponential increase in mortality with age).

### 3.4 Mean Generation Time

We estimated adult apparent survival to be 77.2% (75.2%–79.0% CI) and mean generation time to be 7.38 years, assuming age at first reproduction to be 3 years.

## 4 Discussion

To our knowledge, we have documented the oldest known wild ranid frog, a 21‐year‐old male *R. muscosa* . Despite most populations having few individuals (Figure 3), we estimated longevity at 9.5 years for *R. muscosa* using data from six sites. Previously, the oldest documented wild ranid frog was an 18‐year‐old female European frog ( *Rana temporaria* ) from subarctic Finland (Patrelle et al. 2012). Peralta‐García et al. (2022) reported a lifespan of 9–13 years for wild California red‐legged frogs ( *Rana draytonii* ) from Baja California, México. In contrast to skeletochronology methods used by Patrelle et al. (2012), our methodology, where every newly captured adult frog was assigned the age of three regardless of size, is likely to produce estimates of longevity that are biased low. For example, our oldest frog (21 years) was 65 mm SUL at first capture; 15 mm larger than what is conservatively considered adult size and ~5 mm longer than the average adult male. Thus, our oldest frog was captured at a size that suggests it could have been greater than or equal to 5 years old at first capture, rather than our assumption of 3 years old for all first captures, and therefore, this animal could have plausibly been several years older than 21. Our estimate for longevity of 9.5 years, and even the potential of 11.5 years, is within the range reported for other *Rana* species (6.1–13.4 yr; Reinke et al. 2022). Our calculations of lifespan potentially reaching up to twice as many years suggest that *R. muscosa* is one of the longer‐lived species within this genus.

We did not find any significant difference in survival rates between male and female *R. muscosa* in contrast to other ranid frogs where differences between sexes have been reported (Zhang et al. 2023). Although results were inconsistent, our analyses suggested there may be differences in mortality rates between males and females. At two of three sites where we assessed mortality rates, males had a slower rate of increased mortality with age compared to females and had longer lifespans, yet at the third site, males had a higher rate of increased mortality with age (Table 2, Figure 6). These differences could mirror differential susceptibility to pathogens and subsequent mortality (e.g., *Batrachochytrium dendrobatidis*, Bd, Adams et al. 2017). Alternatively, these differences could reflect an influence of sex chromosomes on aging (Cayuela et al. 2022) or the higher cost of gamete production in females. Further research could examine the extent to which these mortality patterns are influenced by varying Bd prevalence, Bd intensity, sex chromosome differences, or gamete production.

Our generation time estimate (7.48 years) is similar to estimates from genetic assessments of generation time (4–8 years based on archived museum tissue and swabs [Byrne et al. 2024]). Both our values and Byrne et al.'s (2024) values indicate that about seven generations passed since the bottleneck event in *R. muscosa* populations described in the late 1960s (Byrne et al. 2024). Our generation time estimate is greater than Mancini et al. (2025), who estimated generation time of *R. muscosa* at 5.56 years and a mean for all *Rana* species at 5.44 (± 0.18 SE). However, our estimate of generation time (Equation 1) assumes that both fecundity and survival are constant, although amphibian survival can differ across time and space (Morrison and Hero 2003). Captive *R. muscosa* fecundity has been observed to decrease with age (Ian Recchio, Los Angeles Zoo and Botanical Gardens, oral communication; Talisin Hammond, San Diego Zoo Wildlife Alliance, written communication), suggesting that fecundity is likely not truly constant, at least in captivity.

Low genetic diversity and genetic bottlenecks in *R. muscosa* populations across southern California (Schoville et al. 2011) have been attributed to population crashes (Byrne et al. 2024). Despite the expected results of genetic bottlenecks (e.g., loss of alleles and heterozygosity [Allendorf 1986] and reduced ability to cope with disease [Luquet et al. 2012]), some *R. muscosa* populations are persisting, raising the question of how? Genetic diversity lost through bottlenecks can be ameliorated when a population of individuals has high longevity (Bradke et al. 2021; Robinson et al. 2021). Increased longevity may imply some disease resistance, resilience to environmental change (Morris et al. 2008; Peralta‐García et al. 2022), and may help to carry genetic variation through bottlenecks (Robinson et al. 2021). Higher longevity may enable skipped breeding (e.g., Muths et al. 2010) where female *R. m* forgo breeding (a costly activity) for one or more years when individuals are unable to acquire adequate body condition to successfully produce eggs (Peralta‐García et al. 2022). Our identification of a minimally 21‐year‐old individual, and 6.5% of captured animals being > 10 years old, indicates a potential in *R. muscosa* for longevity to be one mechanism by which these populations are persisting. These data highlight the importance of understanding the contribution of older age classes to population growth and genetic diversity.

Capturing such a long‐lived individual *R. muscosa* was unexpected given that populations of *R. muscosa* have been declining in southern California for several decades (Jennings and Hayes 1994; Backlin et al. 2013). Our monitoring surveys documented only a few populations and low numbers of individuals within each population, despite recent augmentations (e.g., Dark Canyon, Fuller Mill) and a habitat expansion project at Little Rock Creek. Persistence of this species is likely challenged by low genetic diversity through numerous processes such as increased wildfire activity (Backlin et al. 2013; Goss et al. 2020), prolonged drought (Griffin and Anchukaitis 2014), increased disease prevalence/intensity (i.e., Bd; Voyles et al. 2012, Smith et al. 2017, Hammond et al. 2025), dam creation, and increased isolation due to predation by introduced fish (Knapp et al. 2007; Backlin et al. 2013; USFWS 2018). Despite these challenges, populations of *R. muscosa* have persisted in southern California, albeit in very low numbers. New information about longevity and other life history characteristics presents potential avenues for better understanding population dynamics. More nuanced insight into the role of older animals in these populations may inform novel strategies to enhance persistence and increase numbers of *R. muscosa* , such as including more adult‐focused conservation actions.

## Author Contributions

**Cynthia J. Hitchcock:** conceptualization (supporting), data curation (supporting), writing – original draft (equal), writing – review and editing (equal). **Adam R. Backlin:** conceptualization (equal), data curation (lead), writing – review and editing (supporting). **Amanda R. Goldberg:** data curation (supporting), formal analysis (supporting), visualization (lead), writing – original draft (equal), writing – review and editing (equal). **Sarah K. Thomsen:** data curation (lead), formal analysis (lead), writing – review and editing (supporting). **Erin Muths:** conceptualization (supporting), writing – review and editing (supporting). **Elizabeth A. Gallegos:** conceptualization (supporting), data curation (supporting), writing – review and editing (supporting). **Robert N. Fisher:** conceptualization (equal), funding acquisition (lead), writing – review and editing (supporting).

## Conflicts of Interest

The authors declare no conflicts of interest.

## Supporting information


**Data S1:** Supporting Information.

**Data S2:** Supporting Information.

**Data S3:** Supporting Information.

## Data Availability

The authors have provided all the required data in the Supporting Information—S1. Data will also be publicly available on figshare, DOI: 10.6084/m9.figshare.29656562.
