# Supplementary material for: Longevity, Age‐Specific Survival, and Mean Generation Time of Rana muscosa : Implications for Conservation of Possibly the Longest‐Lived Ranid Frog
Source: Ecol Evol. 2025 Oct 8;15(10):e72213. doi: 10.1002/ece3.72213 (PMC12507727; doi:10.1002/ece3.72213)
Supplement: Supplementary file 1 — Data S1: Supporting Information. [file ECE3-15-e72213-s003.docx]

**Supplemental Information for “Longevity, age-specific survival, and mean generation time of *Rana muscosa*: implications for conservation of possibly the longest-lived Ranid frog”**

Cynthia J. Hitchcock^1^, Adam R. Backlin^1^, Amanda R. Goldberg^1^, Sarah K. Thomsen^2^, Erin Muths^3^, Elizabeth A. Gallegos^1^, and Robert N. Fisher^2^

^1^U.S. Geological Survey, Western Ecological Research Center, Santa Ana, CA 92701-5001, USA

^2^U.S. Geological Survey, Western Ecological Research Center, San Diego, CA 92101-0812, USA

^3^U.S. Geological Survey, Fort Collins Science Center, Fort Collins Science Center, Fort Collins, CO 80526, USA

*Any use of trade, firm, or product names is for descriptive purposes only and does not imply endorsement by the U.S. Government.*

**Supplemental Information**

A number of different major events likely influenced the number of frogs at sites used in this study:

- Restoration via predatory fish removal at Little Rock Creek enabled the frog population to expand both spatially and numerically after 2009.
- The Station Fire occurred at Devils Canyon in 2009 and was followed by a population boom, then crash (zero to few frogs were found 2014–2024; A Backlin, USGS, pers. comm.). Devils burned a second time within this timeframe in the Bobcat Fire in 2020.
- The Bobcat Fire occurred at Little Rock Creek in 2020 and was followed by a population boom (2022–2023), then crash, with twelve adults being found in 2024 and one in 2025 (A. Backlin, USGS, pers. comm.).
- The Bobcat Fire occurred in South Fork Big Rock Creek in 2020, and frog counts were already in the single digits at this time. No frogs were found in 2023, 2024, or 2025 (A. Backlin, USGS, pers. comm.).
- Major drought occurred in the region intermittently, peaking from 2014–2017.
- Chytrid fungus (*Batrachochytrium dendrobatidis*; Bd) screening showed all populations were infected. Some populations may be more naïve to Bd than others as some populations have experienced die-offs and others have not. However, Hammond et al. (2025) suggest that there are no clear predictors of increased Bd loads across all populations.
- Augmentation from the captive population occurred at: Fuller Mill (2013, 2014, 2015, 2017, 2022, 2023, 2024, 2025), Dark Canyon (2016, 2018, 2019, 2020, 2022, 2024, 2025), Devils Canyon (2018), Vincent Gulch (2018, 2020), and South Fork of Big Rock Creek (2018); (A. Backlin, USGS, pers. comm.).

**Table S1**. Life tables computed in BaSTA for *Rana muscosa* captured from 2000–2022 in California, USA. Included in the life table are the following metrics: Nx = number alive at start of interval, Dx = number of deaths in interval, lx = proportion surviving at start of interval, qx = death rate in interval, ex = remaining life expectancy at end of interval.

| Ages | Nx | Dx | lx | qx | ex |
| --- | --- | --- | --- | --- | --- |
| 0 | 912.476 | 0.000 | 1.000 | 0.000 | 3.600 |
| 1 | 949.032 | 362.000 | 1.000 | 0.381 | 2.600 |
| 2 | 597.248 | 222.000 | 0.619 | 0.372 | 2.825 |
| 3 | 377.799 | 89.000 | 0.389 | 0.235 | 3.088 |
| 4 | 289.774 | 76.000 | 0.297 | 0.262 | 2.945 |
| 5 | 214.000 | 56.000 | 0.219 | 0.262 | 2.872 |
| 6 | 158.000 | 52.000 | 0.162 | 0.329 | 2.766 |
| 7 | 106.000 | 30.000 | 0.109 | 0.283 | 2.957 |
| 8 | 76.000 | 15.000 | 0.078 | 0.197 | 2.999 |
| 9 | 61.000 | 15.000 | 0.063 | 0.246 | 2.631 |
| 10 | 46.000 | 13.000 | 0.047 | 0.283 | 2.394 |
| 11 | 33.000 | 11.000 | 0.034 | 0.333 | 2.209 |
| 12 | 22.000 | 7.000 | 0.023 | 0.318 | 2.096 |
| 13 | 15.000 | 7.000 | 0.015 | 0.467 | 1.921 |
| 14 | 8.000 | 3.000 | 0.008 | 0.375 | 2.132 |
| 15 | 5.000 | 2.000 | 0.005 | 0.400 | 2.169 |
| 16 | 3.000 | 2.000 | 0.003 | 0.667 | 2.362 |
| 17 | 1.000 | 0.000 | 0.001 | 0.000 | 5.000 |
| 18 | 1.000 | 0.000 | 0.001 | 0.000 | 4.000 |
| 19 | 1.000 | 0.000 | 0.001 | 0.000 | 3.000 |
| 20 | 1.000 | 1.000 | 0.001 | 0.000 | 2.000 |
| 21 | 0.000 | 0.000 | 0.001 | 1.000 | 1.000 |

**Table S2**. Years when each site was not surveyed.

| Site | Year |
| --- | --- |
| Dark Canyon | 2000, 2001, 2002 |
| Devils Canyon | 2000, 2004, 2019 |
| Fuller Mill | 2000, 2001 |
| Little Rock Creek | 2000 |
| Big Rock Creek |  |
| Vincent Gulch | 2000, 2016, 2018, 2021, 2022 |


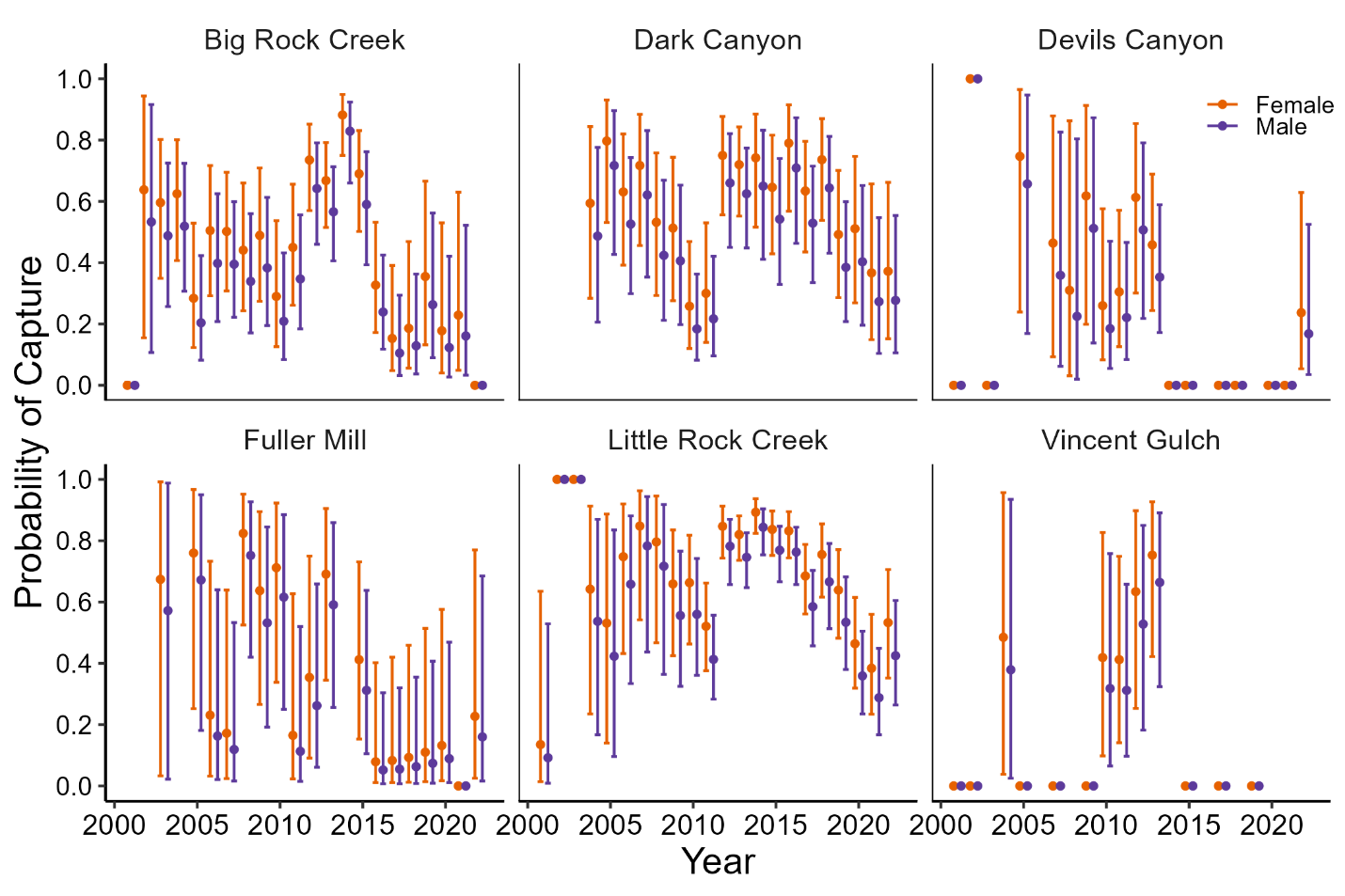


**Figure S1.** Probability of capture (p) for *Rana muscosa* captured between 2000 and 2022 at six sites in southern California. Estimates of p were obtained from top CJS models (Table 3), which included time plus the interaction between sex and site. Probabilities were fixed to 0 when the site was not sampled.
